# Turkish Translation and Cultural Adaptation of the Evaluation in Ayres Sensory Integration (EASI)

**DOI:** 10.1155/oti/6542506

**Published:** 2026-09-22

**Authors:** Aymen Balikci, Ayse Firdevs Aracikul Balikci, Isabelle Beaudry-Bellefeuille, Gamze Cagla Sirma, Fatos Kirteke, Zoe Mailloux, L. Diane Parham, Susanne Smith Roley, Roseann Schaaf

**Affiliations:** ^1^ Sense On Ltd., Istanbul, Türkiye; ^2^ Department of Special Education, Faculty of Education, Istanbul Medipol University, Istanbul, Türkiye, medipol.edu.tr; ^3^ Clínica de Terapia Ocupacional Pediátrica Beaudry-Bellefeuille, Oviedo, Spain; ^4^ Department of Occupational Therapy, Faculty of Health Sciences, Fenerbahce University, Istanbul, Türkiye; ^5^ Faculty of Health Sciences, Department of Physiotherapy and Rehabilitation, Mudanya University, Bursa, Türkiye; ^6^ Jefferson Autism Center of Excellence, Thomas Jefferson University School of Rehabilitation Sciences and Department of Occupational Therapy, Philadelphia, Pennsylvania, USA; ^7^ Department of Pediatrics, Occupational Therapy Graduate Program, University of New Mexico, Albuquerque, New Mexico, USA, unm.edu; ^8^ Collaborative for Leadership in Ayres Sensory Integration, Aliso Viejo, California, USA

**Keywords:** assessment, children, sensory integration

## Abstract

**Introduction:**

Cultural sensitivity and relevance are critical components for best practice in occupational therapy assessment. The Evaluation in Ayres Sensory Integration (EASI) is a performance‐based assessment designed to evaluate sensory perception, sensory reactivity, postural control, ocular control, bilateral integration, and praxis abilities in children aged 3–12 years. Before the development of this tool, no standardized performance‐based assessment of sensory integration functions had included a normative Turkish sample. The objective of this study was to translate and culturally adapt the EASI for Türkiye.

**Method:**

A seven‐step process for translation and cross‐cultural adaptation, grounded in established methodology, was applied. The steps included forward translation, back translation, expert review, linguistic review, cognitive interviews, and pilot testing.

**Results:**

Minor revisions were made following expert and linguistic reviews, but no semantic or conceptual inconsistencies were found in the forward translations. Although some differences emerged in the back translation compared with the original version, these discrepancies did not affect test administration or scoring.

**Conclusion:**

A Turkish version of the EASI has been successfully developed through a systematic translation and cross‐cultural adaptation process. The findings support the linguistic and cultural appropriateness of the Turkish version and provide a foundation for future psychometric validation and research in children in Türkiye.

**Summary**

• The Turkish version of the Evaluation in Ayres Sensory Integration (EASI) demonstrated linguistic and cultural appropriateness through the translation and cross‐cultural adaptation process, providing a foundation for future psychometric validation. Minor linguistic adjustments improved clarity without altering test scoring or administration.
• Therapists and children found the Turkish forms easy to understand.

## 1. Introduction

Sensory integration (SI) is the process in which the brain organizes sensations from the body and environment to form an adaptive response [1–3]. A. Jean Ayres developed the theory and practice of SI, now referred to as Ayres Sensory Integration (ASI) [4]. The theory and practice of ASI has been elaborated upon and further detailed over the past 20 years [5–8].

Adequate SI is essential for successful participation in activities of daily life such as eating, sleeping, dressing, playing, and engaging in leisure activities [7, 9, 10]. Moreover, inefficiencies in SI can contribute to many behavioral, emotional, learning, and socialization problems, which are likely to interfere with successful engagement in daily occupations [11, 12]. Consequently, assessment of SI functions using reliable and valid standardized assessment tools, as well as provision of appropriate intervention programs based on comprehensive assessment of sensory integrative functions, are critical for supporting children′s participation in occupation.

ASI is an evidence‐based occupational therapy intervention that is frequently utilized to support participation of children with many diagnoses, including autism spectrum disorder (ASD), attention deficit hyperactivity disorder (ADHD), and learning disorders (LDs) [2, 8, 11, 13–15]. ASI intervention is a highly individualized approach that addresses the underlying sensory‐motor issues affecting a child′s participation in daily activities at home, at school, or in the neighborhood—such as play, performing chores, doing homework and in‐class assignments, and participating in school or community events.

A comprehensive assessment of SI and related sensory‐motor functions is essential prior to implementation of occupational therapy using Ayres Sensory Integration (OT‐ASI) intervention [16]. Starting in the 1960s, Ayres created a collection of assessments culminating in the Southern California Sensory Integration Tests (SCSITs) [17], later revised and published as the Sensory Integration and Praxis Tests (SIPTs) [18]. These tests and subsequent research including factor analyses provided the foundation for SI constructs and patterns, which include sensory perception, sensory reactivity, postural control, ocular control, bilateral integration, and praxis (cognitive‐motor abilities needed to plan and successfully perform movement activities, particularly when novel actions are required) [18–21]. Since the publication of the SIPT over 25 years ago, there have been limited updates in available assessment for evaluation of SI using a performance‐based, standardized, norm‐referenced assessment tool. Ayres′ early assessments, although clinically useful, did not have the benefit of modern assessment procedures or analyses, and the normative data may not reflect the abilities of children in current populations. Additionally, the normative data were limited to the United States and Canada, without translations to other languages or normative data from additional countries.

The EASI is a recently developed, comprehensive, and accessible set of tests that assess SI and related sensory‐motor functions of children who are 3–12 years old [1, 6, 22]. The EASI tests are the only comprehensive assessment tools currently available with highly diverse international normative data samples from over 80 countries. At the time of this writing, the tests have been translated into more than 25 languages [23, 24].

In this study, we focused on the Turkish translation and cross‐cultural adaptation of the EASI for use in Türkiye while retaining its established conceptual framework and standardized procedures. The adaptation involved examining and refining the translation, administration instructions, scoring procedures, and test materials for conceptual equivalence and cultural relevance to Turkish children. This study did not aim to evaluate the psychometric properties of the Turkish version; rather, it provides a foundation for future reliability and validity studies.

## 2. Materials and Methods

This study followed the International Test Commission (ITC) Guidelines for Translating and Adapting Tests (Second Edition), which describe translation and cross‐cultural adaptation as an initial stage of test adaptation before subsequent empirical evaluation of measurement properties [25]. The methodology was also informed by previously published EASI cultural adaptation studies and other methodological publications on test translation and cross‐cultural adaptation [23–32]. Permission to translate and culturally adapt the EASI into Turkish was obtained from the EASI developers. This study was reviewed and approved by the Clinical Research Ethics Committee of Fenerbahçe University (E‐67888467‐204.01.07‐7405) and conducted in accordance with institutional ethical standards and the principles of the Declaration of Helsinki. All procedures involving human participants were performed in compliance with ethical guidelines, and written informed consent was obtained from the parents or legal guardians of all participating children prior to data collection.

### 2.1. Participants

Nine groups participated in different stages of the translation and cross‐cultural adaptation process. The sample size and composition of the groups involved in the expert committee interview and pretesting phases were informed by previous EASI cultural adaptation research that similarly included professional experts and a small sample of children to evaluate the comprehensibility and cultural appropriateness of the adapted assessment [23]. The characteristics and roles of all participant groups are presented in Table 1.

**Table 1:** Procedures, participants, groups, and their characteristics.

Group	*n*	Participants characteristics	Tasks
1	2	Two native Turkish speakers with advanced proficiency in English. The first individual is an occupational therapy assistant with a strong academic background in the English language and literature, complemented by 5 years of experience as a translator for ASI courses (FT1). The second individual is a highly skilled medical translator with 15 years of professional experience in rehabilitation sciences, specializing in the translation of medical and scientific content (FT2).	The participants in this phase participated exclusively in the forward translation phase. Their role was to independently translate the original English EASI into Turkish.
2	5	Five therapists with substantial expertise in ASI. This includes the first author, who is a physical therapist and occupational therapy student with a PhD in physiology. The first author also serves as a lecturer in the occupational therapy department and has 15 years of clinical experience as an ASI practitioner. The group also includes four occupational therapists, each of whom has over 5 years of specialized experience in pediatric practice and has been involved in assisting ASI training programs for 5 years or more.	The participants in this phase, excluding the first author, participated exclusively in the synthesis phase. Their role was to synthesize the two independent forward translations into a single Turkish version by evaluating linguistic clarity, conceptual equivalence, and cultural appropriateness.
3	2	Two specialists in the Turkish language, one educator with a PhD in educational science and over 15 years of professional experience (second author), and the other is a linguist with expertise in the Turkish language and literature and over 30 years of experience.	The participants in this phase were responsible for the linguistic and cultural review of the Turkish translation. One participant participated exclusively in this phase, whereas the other also participated in the back‐translation review phase.
4	2	A physical therapist who holds a PhD in physical therapy (BT1) and has over 10 years of experience and a medical translator with over 10 years of expertise (BT2).	The participants in this phase participated exclusively in the back‐translation phase and independently translated the Turkish version back into English.
5	4	Four authors on this paper who were also on the research team: An ASI education leader with over 15 years of clinical experience, a licensed physical therapist holding a PhD, and a lecturer in occupational therapy (first author); an ASI‐trained physical therapist with over 10 years of clinical experience and a PhD (fifth author); an ASI‐trained occupational therapist currently pursuing a PhD in occupational therapy, with more than 5 years of clinical experience (fourth author); and an educator with a PhD in educational science and over 15 years of professional experience (second author).	Reviewing the linguistic and semantic compatibility between back translations and the original versions.
6	2	Two authors of the three authors who developed the EASI: Z.M. and S.S.R.	The participants in this phase participated exclusively in the back‐translation review. Their role was to compare the back‐translated English version with the original English EASI, evaluate conceptual equivalence, identify any discrepancies in meaning, and provide feedback and recommendations where necessary.
7	7	Three occupational therapists and four physical therapists all of whom have over 4 years of experience using standardized tests in ASI.	An independent expert panel, whose members participated only in this phase of the study, completed the expert review form and systematically evaluated the translated EASI materials in terms of the clarity and comprehensibility of the Turkish wording, the appropriateness of the terminology, preservation of the intended meaning of the original English version, linguistic and cultural appropriateness, clarity of the administration and scoring instructions of the translated documents, and overall suitability of the Turkish version for future psychometric studies. The experts subsequently discussed the translated materials and suggested minor wording refinements where appropriate.
8	3	Three occupational therapists, trained in the administration of the EASI, with over 5 years of clinical experience with pediatric population.	Testing children to determine practical feasibility and applicability.
9	9	Nine typically developing Turkish children, aged 6–8 years (three females and six males), who were from a private primary school in Istanbul.	Engaged in interviews and offered insights regarding their understanding of the translated materials.

### 2.2. Procedures

We applied the following seven steps for this cultural adaptation study

• Step 1 (forward translation): The two translators in Group 1 were aware of the study objectives and conducted the forward translation of all 20 of the EASI tests. Only one of the 20 EASI tests (Test #11 Praxis: following directions) involves specific verbal instructions for the child. The remaining 19 tests contain examples of verbal directions that may be either read verbatim or slightly reworded by the test administrator to ensure that the child being assessed is understanding the instructions. In addition to the sample verbal instructions for the child, the tests include instructions for administration and scoring for the tester. Both translators independently translated the original English versions of all test sheets and forms into Turkish, with special attention to the Praxis: following directions test, which relies solely on verbal instructions, unlike all other tests in which the examiner may use visual gestures or modeling to explain the instructions for the task. One of the translators with expertise in the English language as well as the ASI literature considered the cultural nuances of English and Turkish to ensure the accuracy of the cultural adaptation. A similar process was applied by Gándara‐Gafo et al. [23].
• Step 2 (synthesis of forms): Group 2 served as the synthesis panel and systematically compared the two independently produced forward translations with the original English version. Through item‐by‐item discussion, the panel reconciled discrepancies and synthesized the two translations into a single preliminary Turkish version. During this process, particular attention was given to conceptual equivalence, linguistic clarity, grammatical accuracy, cultural appropriateness, and the suitability of the terminology for occupational therapy and ASI practice. Alternative wording was discussed whenever necessary to ensure that the translated content accurately conveyed the intended meaning of the original while remaining natural and comprehensible in Turkish. Discussions continued until consensus was reached on every section of the translated materials. The resulting synthesized Turkish version was subsequently submitted to Group 3 for linguistic review and evaluation.
• Step 3 (linguistic review): Group 3 reviewed the Turkish translations for grammatical accuracy, comprehensibility, and cultural appropriateness, providing recommendations for revisions where necessary to enhance linguistic clarity and ensure cultural relevance.
• Step 4 (back translation): Group 4 back‐translated the linguistically revised Turkish versions of the EASI test sheets and forms into English and submitted the back translations to Group 5.
• Step 5 (expert review of the back translation): Groups 5 and 6 (including two of the three original authors of the EASI) jointly reviewed the original English version and the English back‐translated version to evaluate their linguistic and semantic equivalence. When necessary, they identified ambiguous terms or expressions, suggested clarifications, and recommended revisions to ensure consistency with the original version.
• Step 6 (expert committee interview): All EASI documents were checked for potentially necessary corrections or adaptations by ASI trained pediatric occupational and physical therapists (Group 7). The therapists reviewed the tests and offered input to the research team regarding the material′s relevancy. Subsequently, when necessary, they suggested alternative words or phrases to enhance the clarity and understanding of the content. Group 7 first reviewed all translated EASI test sheets, administration instructions, scoring forms, and supporting materials to identify any additional linguistic, professional, or cultural revisions that might be needed. After completing their independent review of the translated materials, each expert independently completed the expert review form. Once all review forms had been completed, the first author convened the experts for a structured in person panel discussion to review the findings, discuss their evaluations, and provide detailed feedback and recommendations. During the panel discussion, the experts considered the clarity and comprehensibility of the translated wording, the appropriateness of the terminology, the preservation of the intended meaning of the original English version, and the cultural suitability of the translated content. Particular attention was given to linguistic or cultural differences in terminology, instructions, administration procedures, and test materials that could potentially affect the interpretation or administration of the adapted assessment. Any differences in opinion were discussed until consensus was reached regarding the proposed revisions. Feedback obtained from both the independently completed expert review forms and the panel discussion was then used to identify issues requiring revision and to refine the Turkish version of the EASI.
• Step 7 (pretesting): Group 8 then conducted pretesting by administering the Turkish version of the EASI to Group 9 (typically developing children aged 6–8 years) to explore the clarity and comprehensibility of the translated instructions and the practicality of the administration procedures. Following the completion of each EASI subtest, the therapists administered the child feedback form, asking children the structured questions and recording their responses. They also asked open‐ended questions to encourage children to describe, in their own words, their understanding of the instructions and to identify any words or directions that were unclear or difficult to understand. Throughout the pretesting process, the therapists documented observational notes regarding the children′s performance, requests for repetition of instructions, and any administration issues encountered. Feedback obtained from the structured child feedback forms, the children′s open‐ended responses, and the therapists′ observational notes was reviewed by the research team. When recurring difficulties or unclear wording were identified, revisions were made to improve the clarity and comprehensibility of the Turkish.

### 2.3. Instruments

#### 2.3.1. The EASI

The EASI is a set of 20 tests, developed for children aged 3–12. Validity and reliability studies have demonstrated good psychometric properties for the EASI, determined by both Rasch and traditional analysis [6, 22, 33–36]. Required testing materials, as well as detailed administration and scoring instructions, are included in the training process, which must be completed by testers prior to application of these tests. The tests were designed to be accessible for children with a wide range of abilities, without undue barriers based on language, cognition, or culture.

#### 2.3.2. Expert Review Form

A structured expert review form was developed by the research team to systematically collect expert feedback during the expert review phase of the Turkish translation and cross‐cultural adaptation of the EASI. The experts used the form to systematically evaluate the translated EASI materials, including the test sheets, administration instructions, scoring forms, and supporting documents. The form was used to assess the clarity and comprehensibility of the Turkish wording, the appropriateness of the terminology, the preservation of the intended meaning of the original English version, and the cultural appropriateness of the translated content. Each item was rated on a 5‐point Likert scale (1 = *strongly disagree*, 2 = *disagree*, 3 = *neutral*, 4 = *agree*, and 5 = *strongly agree*). Experts were also invited to provide written comments and recommendations for revisions. For descriptive analysis, ratings of 4 (*agree*) and 5 (*strongly agree*) were considered positive responses, whereas ratings of 1 (*strongly disagree*), 2 (*disagree*), and 3 (*neutral*) were considered responses indicating that revisions or further consideration might be needed. Frequencies and percentages were calculated for each evaluation item, and the qualitative feedback was used to guide revisions during the translation and cross‐cultural adaptation process.

#### 2.3.3. Child Feedback Form

A structured child feedback form was developed by the research team to systematically collect children′s feedback following the completion of each EASI subtest during the pretesting phase. The form was designed to explore children′s understanding of the translated instructions, the clarity of the language used, and their overall experience while completing each subtest. Children responded to seven statements using a 5‐point Likert scale (1 = *strongly disagree* to 5 = *strongly agree*). Following the child′s responses, the therapist documented additional observations, including whether instructions required repetition, whether any words or instructions caused confusion, and any spontaneous comments made by the child regarding the translated instructions or the testing process. For descriptive analysis, ratings of 4 (*agree*) and 5 (*strongly agree*) were considered positive responses, whereas ratings of 1 (*strongly disagree*), 2 (*disagree*), and 3 (*not sure*) were considered responses indicating that the wording or instructions might require further review. Frequencies and percentages were calculated for each item, and the children′s responses together with the therapists′ qualitative observations were used to guide revisions during the translation and cross‐cultural adaptation process.

### 2.4. Data Analysis

Data were analyzed using descriptive statistics. Frequencies and percentages were calculated to summarize responses obtained from the expert review and the child feedback forms. In addition, qualitative feedback collected throughout each phase of the translation and cross‐cultural adaptation process, including forward translation, synthesis, linguistic review, back translation, expert review, and pretesting, was reviewed by the research team. Comments and recommendations were examined to identify linguistic, conceptual, cultural, and wording issues, and consensus‐based revisions were made at each stage to improve the Turkish version of the EASI. No inferential statistical analyses or psychometric evaluations were performed, as the purpose of this study was limited to the translation and cross‐cultural adaptation of the EASI.

## 3. Results

The results are presented according to the stages of the translation and cross‐cultural adaptation process: (1) forward translation, synthesis of the translations, and reviews of grammatical accuracy and comprehensibility (Steps 1–3); (2) back translation and comparison of the back‐translated version with the original English version (Steps 4–5); and (3) cognitive interviews with professionals and pretesting with children (Steps 6–7). Overall, 34 participants were involved in the study, including translators, rehabilitation professionals, language specialists, educators, members of the research team, the developers of the EASI, therapists, and children.

### 3.1. Results of Steps 1–3: Forward Translation, Synthesis of Forms, and Linguistic Review

Following the forward translation of the EASI documents in Step 1, both translators (100%) reported that the translation process was straightforward and that the original English instructions and terminology were generally easy to translate into Turkish. During Step 2, all members of the synthesis team (100%) reported that the two forward translations were highly similar and sufficiently clear for synthesis. Based on the team′s discussions, only minor wording revisions were considered necessary to improve the clarity of the translated instructions and to standardize the administration procedures for therapists. These revisions were made to enhance the comprehensibility of the Turkish version while maintaining the intended meaning of the original EASI documents. First, Group 2 decided to leave some terms in their original form to guarantee clarity and comprehensibility for therapists. For example, occupational therapists and physiotherapists often use the terms “extension” and “segmentation” during clinical practice. The meaning of these two words is clear and unambiguous for them. Using other terms could be confusing; therefore, it was decided to use these words in the way health professionals in Türkiye use them.

In addition, Group 1 found it difficult to identify precise Turkish equivalents for some phrases and terms, and thus asked Group 2 to determine the most understandable terminology or to identify synonymous terms (e.g., Jogs: Sarsıntı/Sapmalar). Secondly, Group 2 made minor modifications to Groups 1′s translations to improve the children′s or therapist′s comprehension of the instructions (see Table 2 for examples). After the synthesis team prepared the preliminary Turkish version, the translated documents were reviewed by two linguists with expertise in Turkish language and grammar. Both linguists (100%) reported that the Turkish version was generally clear, understandable, and appropriate for its intended audience. However, they also indicated that several minor grammatical, lexical, and stylistic revisions would improve the fluency, naturalness, and readability of the text. Based on their recommendations, wording, sentence structure, punctuation, and terminology were refined where necessary to enhance the clarity and consistency of both the children′s instructions and the therapists′ administration guidelines, while preserving the intended meaning of the original English version. Examples of these revisions are presented in Table 3.

**Table 2:** Examples of translation refinements made during the synthesis process.

Test and item	Original English version	Forward translations	Synthesized Turkish version
Praxis: following directions: 1B	Put both arms up.	FT1: Kollarını kaldır.	Her iki kolunu yukarı kaldır.
		FT2: Her iki kolunu da yukarı kaldır.	
Praxis: following directions: 2H	Make a ball with this piece of paper.	FT1 and FT2: Bu kağıtla bir top yap.	Bu kağıttan bir top yap.
Praxis: sequences: 1F	With mouth closed, sniff (quickly inhale) air in through nose, three times.	FT1: Ağzı kapalı iken 3 kere hızlı şekilde burun içine hava çekilir.	Ağız kapalıyken burnundan hızlı bir şekilde 3 kere hava çek.
		FT2: Ağız kapalı iken, 3 kez hızlı bir şekilde burnunuzdan hava alın (havayı koklamak gibi).	

**Table 3:** Examples of linguistic revisions made during the linguistic review.

Test and/or test item	Original English version	Synthesized Turkish version	Final Turkish translation with linguistic modifications by Group 3
Praxis: following directions	Examiner and child are seated for the entire test, with the table on the side of the examiner′s writing hand.	Testör ve çocuk bütün test boyunca, masa testörün yazdığı elinin tarafında kalacak şekilde otururlar.	Testör ve çocuk test boyunca karşılıklı olacak şekilde oturur. Testörün kolaylıkla not alabilmesi için masa yazdığı elinin olduğu tarafta bulunur.
Praxis: following directions, trial 1B	Listen to what I say. Wait until I finish talking and then you do it: Put your hand on your head.	Söylediğimi dinle. Benim konuşmam bitene kadar bekle ve sonra sen yap: Ellerini başının üstüne koy.	Beni dinle, konuşmam bitene kadar bekle ve söylediğimi yap.
Praxis: ideation	Ask child to sit in a child sized chair next to a table.	Çocuğu, boyutlarına uygun bir sandalyede masanın yanında oturtun.	Çocukla boyutlarına uygun bir sandalyede olacak şekilde masaya karşılıklı oturun.
Ocular: motor and praxis, ocular stabilization, trial	Hold eraser end of pencil at child′s eye level midline, and about 12 ^″^ (30 cm) in front of child′s face, and say, Watch me. This time I′m moving my head, but I am keeping my eyes on the eraser.	Kalemin silgili ucunu çocuğun yüzünün önünde, orta hatta çocuğun göz seviyesinden 30 cm uzakta tutun ve şöyle söyleyin: “Beni izle. Bu sefer başımı hareket ettiriyorum ama gözlerimi silgi üzerinde tutuyorum.”	Testör, silginin ucu çocuğun göz hizasında ve orta hatta, çocuğun yüzüne yaklaşık 30 cm uzaklıkta olacak şekilde tutar. “Beni izle. Şimdi başımı hareket ettiriyorum ama gözlerimi silgiden ayırmıyorum.” der.

### 3.2. Results of Steps 4 and 5: Back Translation and Comparison With the Original English Version

All participants in Group 5 (100%) reported that no substantial differences were identified in the semantic meaning or conceptual content between the back‐translated English versions and the original English version. Overall, the back translation accurately reflected the original EASI documents, indicating that the intended meaning had been preserved during the translation process. The items of the “Praxis: following directions” test were generally consistent with the original English version. Nonetheless, modifications made to the instructional components of several tests to improve the clarity and comprehensibility of the Turkish version resulted in some discrepancies between the back‐translated version and the original English documents (see Table 4 for examples). All participants in Group 6 (100%) examined these discrepancies in detail and agreed that they reflected intentional modifications made to enhance the clarity of the Turkish translation rather than changes in meaning. The experts concluded that these revisions remained sufficiently consistent with the original English version and appropriately preserved the intended meaning of the EASI items and administration instructions.

**Table 4:** Examples of comparisons between the back translation and the original English version.

Test	Original English version	Turkish version after linguistic review	Decided back translation
Praxis: positions	Child may assume position either mirrored or nonmirrored, that is, no penalty for nonmirrored responses.	Çocuk gösterilen pozisyonu aynada görüldüğü gibi (tam tersi) ya da aynı şekilde yapabilir (her iki şekilde de puan alır).	The child may replicate the shown position either as a mirror image (reversed) or exactly as demonstrated (both are awarded points).
Tactile perception: localization	Now show me where you feel me touch without looking.	Şimdi bakmadan nerene dokunduğumu bana göster.	Now show me where I touched you without looking.
Bilateral integration, scoring, trial	Examiner presents trial by tapping palm of hand against thigh at speed of about 1 s per each “section” of pattern.	Testör, her bir madde için 1 saniyelik hızla olacak şekilde elinin avuç içini bacağına vurarak yönergeyi sunar.	The examiner presents the instruction by tapping the palm of their hand against their thigh at a speed of 1 s for each item.

### 3.3. Results of Steps 6 and 7: Cognitive Interviews and Pretesting of the Turkish Version of the EASI

During the expert review phase, all participants in Group 7 completed the expert review form and participated in structured discussions regarding the translated EASI materials. Across all evaluation items, 100% of the experts rated the translated materials as either 4 (*agree*) or 5 (*strongly agree*). Specifically, all experts agreed that the Turkish wording was clear and understandable; the instructions could be easily understood by Turkish children and therapists where applicable; the terminology was appropriate for occupational therapy and ASI practice; the translated text was linguistically natural in Turkish; the intended meaning of the original English version had been preserved; the translated expressions were culturally appropriate and contained no culturally confusing or inappropriate wording; the administration and scoring instructions were clear for therapists; and the translated documents were internally consistent and suitable for use in future psychometric studies.

During the discussions, the experts noted that most of the translated materials consisted of administration instructions, scoring procedures, and documentation intended for therapists. Accordingly, they considered the final Turkish version to be highly understandable and appropriate for professional use, with only minor recommendations to improve the clarity of a small number of expressions. The Praxis: following directions subtest, which includes verbal instructions directed to children, was reviewed in particular. All experts (100%) agreed that these child‐directed instructions were clear, comprehensible, and appropriate for the target age group. The few suggestions provided focused on improving the wording of specific instructions to enhance clarity rather than addressing conceptual or cultural concerns, and these revisions were incorporated into the final Turkish version. Group 8 reported that they did not experience any difficulties related to the clarity of the translated materials or the administration and scoring procedures while administering the Turkish version of the EASI. All three therapists (100%) indicated that the translated administration instructions, scoring criteria, and test forms were clear and easy to follow throughout the assessment. The only observation reported by the therapists was that the extensive written instructions required careful attention and additional time during administration; however, they considered this to reflect the comprehensive nature of the EASI rather than any issue related to the Turkish translation or cross‐cultural adaptation.

Consistent with the therapists′ observations, all children (100%) provided positive ratings (*agree* or *strongly agree*) for all items evaluating the clarity and comprehensibility of the translated materials. Specifically, all children reported that they understood what they needed to do, found the therapist′s directions easy to understand, considered the therapist′s wording easy to understand, knew what to do throughout the assessment, and did not find any of the instructions confusing (see Table 5). All children also reported that they enjoyed completing the assessment, whereas seven children (77.8%) indicated that they would be willing to complete the assessment again and two children (22.2%) responded “not sure.”

**Table 5:** Children′s feedback on the clarity and comprehensibility of the Turkish EASI during pretesting (*n* = 9).

Child feedback form item	Strongly agree (5) *n* (%)	Agree (4) *n* (%)	Not sure (3) *n* (%)	Disagree (2) *n* (%)	Strongly disagree (1) *n* (%)
1. I understood what I needed to do.	4 (44.4%)	5 (55.6%)	0	0	0
2. The therapist′s directions were easy to understand.	4 (44.4%)	5 (55.6%)	0	0	0
3. The words the therapist used were easy to understand.	5 (55.6%)	4 (44.4%)	0	0	0
4. I always knew what to do during the test.	4 (44.4%)	5 (55.6%)	0	0	0
5. Nothing the therapist said was confusing.	3 (33.3%)	6 (66.7%)	0	0	0
6. I enjoyed doing this test.	1 (11.1%)	8 (88.9%)	0	0	0
7. I would like to do this test again.	1 (11.1%)	6 (66.7%)	2 (22.2%)	0	0

The therapists reported that none of the children required clarification because of unclear wording, asked about the meaning of any translated word, or appeared to misunderstand the translated instructions during administration. Likewise, no therapist observed that any instruction was difficult for the children to understand. During the open‐ended interviews, several children commented that the assessment was easy to understand but took a long time to complete and was somewhat tiring. The research team interpreted these comments as reflecting the comprehensive nature and duration of the EASI rather than any issues related to the Turkish translation or cross‐cultural adaptation.

The results of this study included successful completion of a seven‐step Turkish translation and cultural adaptation process for the EASI, as recommended in the literature [23, 24, 26, 27, 29].

## 4. Discussion

The aim of this study was to translate and culturally adapt the EASI for use in Türkiye through a systematic, multistep cross‐cultural adaptation process. Overall, findings from the translation, back translation, expert committee evaluation, and pretesting processes supported the conceptual, semantic, linguistic, and cultural appropriateness of the Turkish version relative to the original English EASI. Only minor wording refinements were required to improve clarity and comprehensibility, and no signs, symbols, materials, or task demonstrations were identified as culturally inappropriate, ambiguous, or likely to be interpreted differently in the Turkish context. These findings are consistent with the international development of the EASI, which incorporated input from SI experts from diverse cultural backgrounds to promote its cross‐cultural applicability [22–25]. However, the present findings should not be interpreted as evidence of psychometric or measurement equivalence between the Turkish and original versions, as these properties were beyond the scope of this study and require further empirical evaluation.

In the present study, the two independent forward translations of the EASI demonstrated a high degree of semantic and conceptual equivalence, allowing the synthesis panel to produce a unified Turkish version with only minor modifications. The differences identified during the synthesis process were primarily related to terminology preferences, wording style, and linguistic refinements rather than conceptual discrepancies that affected the meaning of the original items. This finding may be attributed to the translators′ experience in English translation and their familiarity with SI and rehabilitation terminology. Previous guidelines for translation and cross‐cultural adaptation emphasize that translators with subject‐specific knowledge are better equipped to preserve conceptual equivalence and select terminology that is both accurate and culturally appropriate [26–32]. The subsequent linguistic review further enhanced the quality of the translated version by improving readability, linguistic naturalness, and comprehensibility. Revisions at this stage included refining wording, replacing less familiar expressions with more commonly used Turkish terminology, and rephrasing instructions to make them clearer for users while maintaining the intended meaning of the original items. These refinements are consistent with cross‐cultural adaptation frameworks, which emphasize that successful adaptation extends beyond literal translation and requires semantic, idiomatic, experiential, and conceptual equivalence across languages and cultures [26–31]. Likewise, Geisinger [37] highlighted that even minor linguistic modifications, such as clarifying wording and rephrasing instructions, can substantially improve comprehensibility and reduce measurement bias. The findings of the present study are also consistent with previous Spanish [23] and Swedish [24] adaptations of the EASI, which similarly required only minor linguistic refinements during the cross‐cultural adaptation process.

The back translation and comparison with the original English version confirmed that the Turkish version preserved the conceptual meaning and intent of the EASI despite a small number of linguistic differences. The discrepancies identified during this stage were primarily attributable to explanatory examples and wording refinements that had been intentionally introduced to improve clarity, comprehensibility, and cultural appropriateness rather than to conceptual inconsistencies. This finding is consistent with the cross‐cultural adaptation literature, which recognizes that differences between the original and back‐translated versions are often expected when culturally appropriate modifications are made to achieve functional and conceptual equivalence rather than literal correspondence [37–40]. As noted by Behr [39], back translation may generate “false alarms,” whereby intentional linguistic adaptations appear as discrepancies despite preserving the intended meaning of the original instrument. In the present study, the comments provided by the EASI developers indicated that most suggested revisions concerned the administration manual, particularly the test preparation procedures and examiner instructions. These recommendations primarily focused on improving clarity, consistency, and alignment with the standardized administration of the assessment rather than altering the underlying concepts of the test. In contrast, for the Praxis: following directions subtest, greater emphasis was placed on ensuring that the Turkish translation accurately conveyed the intended meaning and purpose of the original verbal instructions, given that children′s performance depends directly on their interpretation of these directions. Accordingly, comparison of the back‐translated and original versions focused on preserving conceptual equivalence while maintaining the standardized intent of the assessment, rather than achieving a literal word‐for‐word translation [38–40].

The expert interview findings indicated a high level of agreement regarding the clarity, comprehensibility, cultural appropriateness, and conceptual equivalence of the Turkish EASI. An important strength of this phase was that all participating experts had theoretical knowledge of ASI and experience with standardized ASI assessments. This background may have facilitated the evaluation process by enabling the experts to distinguish between linguistic issues and the underlying theoretical constructs of the assessment, resulting in more consistent and clinically meaningful feedback [25, 39, 40]. Similar emphasis on involving professionals with ASI knowledge has been reported in previous EASI adaptation studies [23, 24]. In the Swedish adaptation of the EASI, occupational therapists with ASI training participated throughout the translation and expert review process because their theoretical and clinical knowledge was considered essential for evaluating semantic, experiential, and conceptual equivalence beyond linguistic accuracy [24].

Similarly, the pretesting phase of the present study benefited from the involvement of occupational therapists who had received EASI training and were familiar with both the theoretical principles of ASI and the standardized administration procedures of the assessment. Their familiarity with the EASI may have facilitated the identification of any remaining issues related to the clarity and usability of the Turkish version during administration, while minimizing the possibility that administration‐related issues would be interpreted as translation problems [25, 28, 40]. Therapists consistently reported that the translated materials were easy to understand and administer, and the participating children generally experienced no difficulty understanding the test instructions. These findings are consistent with the Spanish cultural adaptation study, in which both occupational therapists and children reported good overall comprehension of the translated materials, with only a few revisions required following the cognitive interviews [23].

The ease with which children understood the test instructions may also reflect the design of the EASI itself. In most subtests, the detailed administration instructions are directed primarily to the examiner rather than to the child. Consequently, the linguistic quality of the translated materials is particularly important for therapists, whereas relatively few verbal instructions require direct child comprehension [22–25]. The principal exception is the Praxis: following directions subtest, in which successful performance depends on accurately understanding spoken instructions [22, 28]. Consistent with the Spanish adaptation, this was the language‐dependent subtest that required the greatest attention during the cultural adaptation process [23].

Although some children demonstrated reduced attention during administration, this observation was more likely related to the overall duration of the assessment than to difficulties associated with the Turkish translation or cultural adaptation.

Building on these findings, it is also necessary to recognize the limitations of the present study. Limitations in this study include that we employed convenience samples of occupational and physical therapists and typically developing children for the comprehensibility interviews, with the child pretesting involving a relatively small sample (*n* = 9) recruited from a single private primary school in Istanbul and limited to a narrow age range (6–8 years), although the EASI is designed for children aged 3–12 years. These sampling characteristics may have introduced some bias; nonetheless, we believe these participants adequately represented the populations that will utilize or be assessed with these tests in Türkiye. In addition, this cultural adaptation occurred using the current version of the EASI. Since final psychometric analyses are underway, it is likely that some items will be dropped, which may, in fact, eliminate some of the items that require minor adjustments. Future psychometric research on the final translated Turkish version of the EASI, with occupational therapists who are trained in and have utilized the EASI for an extended period, is highly suggested and planned.

Despite these limitations, this study has important implications for occupational therapy practice and research in Türkiye. Occupational therapists in Türkiye have had limited access to comprehensive, performance‐based assessments for evaluating SI and praxis, particularly in young children. As highlighted in our previous work, the absence of appropriate standardized assessments has limited the comprehensive evaluation of SI functions in this age group [41]. The availability of a culturally adapted Turkish version of the EASI represents an important step toward addressing this gap by providing clinicians and researchers with access to a comprehensive assessment of SI functions. However, construct, method, and item equivalence were not quantitatively evaluated in the present study, and further research is needed to examine these aspects together with the reliability, validity, and other relevant psychometric properties of the Turkish EASI before conclusions can be drawn regarding its psychometric performance in Turkish children.

## 5. Conclusions

The findings of this study support the linguistic, conceptual, and cultural appropriateness of the Turkish version of the EASI following a systematic translation and cross‐cultural adaptation process. The adapted version incorporates the revisions identified through expert review and pretesting and provides a foundation for future studies evaluating its psychometric properties and clinical applicability in Turkish children.

## Author Contributions

Paper conceptualization: A.B., F.K., Z.M., L.D.P., S.S.R., R.S., and I.B‐B. Methodology: A.B., F.K., and I.B‐B. Validation: A.B., Z.M., F.K., and I.B‐B. Formal analysis: A.B., Z.M., F.K., and I.B‐B. Investigation: A.B., A.F.A.B., and F.K. Resources: A.B., F.K., G.C.S., and A.F.A.B. Data curation: A.B., F.K., G.C.S, and A.F.A.B. Writing—original draft preparation: A.B., F.K., Z.M., L.D.P., S.S.R., R.S., and I.B‐B. Writing—review and editing: A.B., F.K., Z.M., L.D.P., S.S.R., R.S., and I.B‐B. Visualization: A.F.A.B. and G.C.S. Supervision: L.D.P., S.S.R., R.S., Z.M., and I.B‐B. Project administration: A.B. and F.K.

## Funding

No funding was received for this manuscript.

## Disclosure

All authors have read and agreed to the published version of the manuscript. A preprint has previously been published [1].

## Ethics Statement

The study was conducted in accordance with the Declaration of Helsinki and approved by the Clinical Research Ethics Committee of Fenerbahce University (E‐67888467‐204.01.07‐7405).

## Consent

Informed consent was obtained from all caregivers involved in the study.

## Conflicts of Interest

The authors declare no conflicts of interest.

## Supporting information


**Supporting Information** Additional supporting information can be found online in the Supporting Information section. File S1: Completed Test Adaptation Reporting Standards (TARES) checklist.

## Data Availability

The data that support the findings of this study are available from the corresponding author upon reasonable request.
