# Supplementary material for: Turkish Translation and Cultural Adaptation of the Evaluation in Ayres Sensory Integration (EASI)
Source: Occup Ther Int. 2026 Sep 22;2026:6542506. doi: 10.1155/oti/6542506 (PMC13595813; doi:10.1155/oti/6542506)
Supplement: Supplementary file 1 [file OTI-2026-6542506-s001.pdf]

Completed Test Adaptation Reporting Standards (TARES) Checklist

Turkish Translation and Cultural Adaptation of the Evaluation in Ayres Sensory Integration® (EASI)

Reporting framework and scope note. This checklist was completed using the TARES reporting framework (Iliescu et al., 2023/2024) for test adaptation studies. The present study was designed specifically as a translation and cross-cultural adaptation study, not as a psychometric validation or norming study. Accordingly, TARES items requiring quantitative measurement-equivalence, reliability, internal-structure, relations-to-other-variables, testing-consequence, or norming analyses are marked as not applicable to the present study aim or as not quantitatively evaluated, with the reason stated transparently. This approach follows TARES guidance that unavailable forms of evidence should be explicitly identified rather than implied or manufactured.

TARES reference: Iliescu, D., Bartram, D., Zeinoun, P., Ziegler, M., Elosua, P., Sireci, S., Geisinger, K. F., Odendaal, A., Oliveri, M. E., Twing, J., & Camara, W. (2024). The Test Adaptation Reporting Standards (TARES): Reporting test adaptations. International Journal of Testing, 1–23. https://doi.org/10.1080/15305058.2023.2294266

Category	ID	Checklist item	Status for this manuscript	Manuscript location	Completed response / rationale
Title and abstract	1a	Title	Reported	p. 1	The title identifies the EASI and explicitly identifies the work as Turkish translation and cultural adaptation.
Title and abstract	1b	Abstract	Reported	p. 2	The abstract identifies the EASI, target context (Türkiye), adaptation design, main procedures, results, and conclusion.
Introduction	2a	The test	Reported	pp. 3–4	The EASI is introduced as a comprehensive performance-based assessment of sensory integration and related sensory-motor functions.
Introduction	2b	Intended population	Reported	pp. 2, 4	The intended population is children aged 3–12 years; the target context is Türkiye.
Introduction	2c	Purpose and intended use	Reported	pp. 3–5	The constructs/domains assessed and the intended occupational therapy assessment context are described.
Introduction	2d	Copyright	Addressed within study scope	p. 5	Permission to translate and culturally adapt the EASI into Turkish was obtained from the EASI developers. The manuscript does not make a separate claim regarding ownership of the adapted version.
Introduction	2e	Need for adaptation	Reported	pp. 2–4	The manuscript explains the lack of an appropriate standardized performance-based sensory integration assessment with a Turkish normative context and the resulting clinical/research gap.
Introduction	2f	Appropriateness of adaptation	Reported	pp. 4–5	The manuscript explains that adaptation was selected to retain the established EASI conceptual framework and standardized procedures while addressing Turkish linguistic and cultural requirements.
Introduction	2g	Coverage of adaptation	Reported	p. 9	All 20 EASI tests were included in forward translation; the manuscript describes translation of all test sheets and forms.

Category	ID	Checklist item	Status for this manuscript	Manuscript location	Completed response / rationale
Introduction	2h	Likelihood of biases	Reported qualitatively	pp. 9–11, 22, 26–27	Potential linguistic/cultural sources of interpretation or administration bias were considered during translation and expert review. Quantitative construct/method/item bias analyses were outside the study scope and are explicitly identified as future work.
Methods – Translation	3a	Translators	Reported	pp. 5–8; Table 1	Numbers, professional backgrounds, language expertise, EASI/ASI expertise, and roles of translators/reviewers are described.
Methods – Translation	3b	Translation design	Reported	pp. 9–12	A seven-step process is described: forward translation, synthesis, linguistic review, back-translation, expert review of back-translation, expert committee interview, and pre-testing.
Methods – Translation	3c	Similarity and suitability of test components	Reported	pp. 9–12, 19–21	Instructions, terminology, administration/scoring procedures, materials, and child comprehension were evaluated through expert judgment and pre-testing; resulting revisions are reported.
Methods – Materials	4a	Test stimuli	Reported	pp. 9–11, 22	Test sheets/materials and possible cultural issues in signs, symbols, materials, and demonstrations were reviewed; no culturally inappropriate elements were identified.
Methods – Materials	4b	Instructions and scoring rubrics	Reported	pp. 9–12, 19–21	Administration instructions, scoring instructions/forms, and translated wording were explicitly reviewed and pre-tested.
Methods – Materials	4c	Test manual	Not separately applicable to this study	pp. 9–12	The study adapted the EASI test sheets/forms, administration and scoring instructions, and supporting materials. A separate new Turkish test manual was not an outcome evaluated in this translation/adaptation study.
Methods – Materials	4d	Test reports	Not applicable	—	No test-report product or automated/clinical report was developed or adapted as part of this study.
Methods – Materials	4e	Training materials	Not separately evaluated	p. 12	The manuscript notes that required materials and detailed administration/scoring instructions are included in the EASI training process. Adaptation of a separate training program/material set was not an objective of this study.
Methods – Materials	4f	Testing conditions	Addressed within study scope	pp. 11–12, 19–21	Standardized administration procedures and their practicality were reviewed during pre-testing; administration issues and requests for repetition were documented.
Methods – Participants/Sample	5a	Sample size	Reported	pp. 5–8; Table 1; p. 14	Sample sizes are provided for all nine participant groups; 34 participants were involved overall. The expert/pre-test group structure was informed by prior EASI adaptation research.
Methods – Participants/Sample	5b	Sample collection procedure	Reported	pp. 5–8, 11	Participant groups and their roles are described; the child pre-test sample and expert groups are identified, with ethics/consent procedures reported.
Methods – Participants/Sample	5c	Sample composition	Reported	pp. 5–8; Table 1	Relevant professional expertise is described for expert groups; the child group is described by

Category	ID	Checklist item	Status for this manuscript	Manuscript location	Completed response / rationale
					developmental status, age and other available characteristics in Table 1.
Methods – Participants/Sample	5d	Sample relevance	Reported with limitations	pp. 5–8, 25–27	The relevance of ASI/EASI-trained professionals and children to comprehensibility/practical administration is explained; limitations of convenience sampling, n=9, single school and narrow age range are acknowledged.
Results – Equivalence	6a	Construct equivalence	Scope-limited / no quantitative equivalence testing	pp. 19–22, 27	Expert review, back-translation and pre-testing provide judgmental/conceptual adaptation evidence. Quantitative construct equivalence/invariance was not evaluated; this is explicitly stated as beyond scope.
Results – Equivalence	6b	Method equivalence	Not quantitatively evaluated	pp. 22, 27	Administration procedures were qualitatively reviewed, but formal quantitative method-equivalence analysis was not conducted. The limitation is explicitly stated.
Results – Equivalence	6c	Item equivalence	Not quantitatively evaluated	pp. 22, 27	Item-level wording was reviewed during adaptation, but DIF or other quantitative item-equivalence analyses were not conducted. The limitation is explicitly stated.
Results – Equivalence	6d	Limits of equivalence	Reported	pp. 22, 26–27	The manuscript explicitly states that findings support linguistic/conceptual/cultural appropriateness but should not be interpreted as psychometric or measurement equivalence.
Results – Equivalence	6e	Solutions for non-equivalence	Not applicable at this stage	pp. 14–21	Linguistic/cultural issues identified during adaptation were resolved by consensus-based revisions. No quantitative non-equivalence was tested or established because psychometric equivalence testing was outside scope.
Results – Reliability	7a	Reliability	Not applicable to present study aim	pp. 5, 14, 27	No reliability analysis of the Turkish version was performed. The manuscript explicitly states that psychometric evaluation was not an aim and identifies reliability as future research.
Results – Validity	8a	Validity evidence	Scope-limited adaptation evidence only	pp. 14, 19–22, 27	The study provides translation, expert-review and pre-test evidence relevant to linguistic/conceptual/cultural appropriateness, but does not claim a full psychometric validity argument for Turkish EASI scores.
Results – Validity	8b	Validity evidence based on test content	Addressed qualitatively	pp. 9–12, 19–22	Experts reviewed terminology, intended meaning, cultural appropriateness, instructions, scoring forms and supporting materials; revisions and agreement are reported.
Results – Validity	8c	Validity evidence based on response processes	Addressed qualitatively	pp. 11–13, 20–21	Children were asked to explain their understanding of instructions; structured feedback, open-ended responses and therapist observations were used to identify comprehension problems.
Results – Validity	8d	Validity evidence based on internal structure	Not applicable to present study aim	pp. 5, 14, 27	No factor-analytic/internal-structure analysis of the Turkish version was performed; psychometric evaluation was explicitly outside scope.

Category	ID	Checklist item	Status for this manuscript	Manuscript location	Completed response / rationale
Results – Validity	8e	Validity evidence based on relations to other variables	Not applicable to present study aim	pp. 5, 14, 27	No convergent, discriminant, criterion or other relations-to-variables analyses were performed because the study was limited to translation and cultural adaptation.
Results – Validity	8f	Validity evidence based on testing consequences	Not evaluated in this adaptation study	pp. 5, 14, 27	Testing consequences were not empirically evaluated. Clinical/psychometric use is appropriately deferred pending future validation.
Results – Norms	9a	Norming procedure	Not applicable to present study aim	pp. 4–5, 14, 27	No Turkish norming procedure was conducted; the study did not aim to estimate psychometric properties or norms.
Results – Norms	9b	Norm tables	Not applicable	—	No Turkish norm tables were generated in this translation and cultural adaptation study.
Discussion	10a	Practical relevance	Reported	pp. 26–27	The discussion explains the clinical/research gap in Türkiye and the practical relevance of a culturally adapted Turkish EASI.
Discussion	10b	Theoretical relevance	Reported to the extent relevant	pp. 22–25	Findings are discussed in relation to EASI international development and cross-cultural adaptation literature; no broader theoretical claims beyond the adaptation evidence are made.
Discussion	10c	Limitations	Reported	pp. 22, 26–27	Sampling limitations, age-range limitation, current-version limitation, and the absence of quantitative construct/method/item equivalence and psychometric evaluation are explicitly acknowledged.
Discussion	10d	Future research	Reported	pp. 26–27	Future reliability, validity, measurement-equivalence and other psychometric evaluation of the Turkish EASI are explicitly recommended.
Supplementary	11a	Registration	Reported	p. 28	The manuscript explicitly states: “This study was not preregistered.”
Supplementary	11b	Funding	Reported	p. 28	The manuscript explicitly states that the research received no external funding.

Author note for submission. Items marked “not applicable,” “not quantitatively evaluated,” or “scope-limited” reflect the predefined scope of the study rather than missing analyses from a psychometric validation study. The revised manuscript now explicitly states this scope in the Introduction/Methods and identifies the limits of equivalence and the need for subsequent reliability, validity, measurement-equivalence, and other psychometric studies in the Discussion.
